# Genetics and Opioids: Towards More Appropriate Prescription in Cancer Pain

**DOI:** 10.3390/cancers12071951

**Published:** 2020-07-18

**Authors:** Dario Bugada, Luca F. Lorini, Roberto Fumagalli, Massimo Allegri

**Affiliations:** 1 Emergency and Intensive Care Department, ASST Papa Giovanni XXIII, 24127 Bergamo, Italy; llorini@asst-pg23.it; 2 Italian Pain Group; allegri@italianpaingroup.com; 3 School of Medicine and Surgery, University of Milan-Bicocca, 20900 Monza, Italy; roberto.fumagalli@unimib.it; 4 Department of Anesthesiology, ASST Grande Ospedale Metropolitano Niguarda, 20162 Milan, Italy; 5 Pain Therapy Service—Fondazione Policlinico Monza, 20900 Monza, Italy

**Keywords:** pain, genetics, opioids, cancer pain, personalized medicine, genomics

## Abstract

Opioids are extensively used in patients with cancer pain; despite their efficacy, several patients can experience ineffective analgesia and/or side effects. Pharmacogenetics is a new approach to drug prescription based on the “personalized-medicine” concept, i.e., the ability of tailoring treatments to each individual’s genetic/genomic profile. Pharmacogenetics aims to identify specific genetic variants that influence pharmacokinetics and pharmacodynamics of drugs, better determining their effectiveness/safety profile. Opioid response is a complex scenario, but some gene variants have shown a correlation with pain sensitivity, as well as with opioid metabolism and clinical efficacy/adverse events. Although questions remain unanswered, some of these gene variants may already be used to identify specific patients’ phenotypes that are more prone to experience better clinical response (i.e., better analgesia and/or less adverse events). Once adopted, this approach to opioid prescription may improve a patient’s outcome. This review summarizes the available data on genetic variants and opioid response: we will focus on basic pharmacogenetic and its impact in the clinical scenario discussing how they may lead to more appropriate opioid prescription in cancer patients.

## 1. Introduction

Opioids have been used for years in chronic pain management, both in cancer and non-cancer patients. Even though they are effective in many cases, opioids have some important side effects, which limit their effectiveness in clinical practice. Adverse events such as constipation, nausea, vomiting, itching, and sedation can occur also, even after a single dose. Long-term administration can lead to tolerance and hyperalgesia, reducing effectiveness despite the increasing doses and molecule rotation [1,2]. The majority of adverse events disappears with time (tolerance), while some of them, such as ileus and itching, does not have any tolerance effect, further limiting the efficacy of opioid therapy. As well, long-term use of opioids may influence the immune system (by reducing immune competence [3]) and the endocrine system (by creating hypogonadism and hypoadrenalism [4]). Finally, the over-prescription and abuse of opioids for pain in the last decade has led to the opioid crisis in North America. This epidemic and the consequent efforts to control it are leading to strong limitations to the provision of cancer pain relief, as well: new knowledge and new tools are required to improve opioid prescription, providing safe and effective pain control without increasing the incidence of substance abuse disorders [5]. 

As well as for most of the drugs (probably even more for opioids) it is difficult to find the optimal combination between appropriate dose and side effects. The concept of the so-called “personalized medicine” could help to overcome this problem, offering new information to find the best effective and safest dose for a specific patient and to provide maximum efficacy with no/minimum adverse events. The philosophy of “personalized medicine” is to choose medications according not only to patient’s history but also to his genetically determined pharmacokinetic and pharmacodynamics. In fact, the major determinant in defining the specificity of each individual is the genetic profile, which defines any aspect of pharmacology and physiology, including the response to pain medication. Great attention has been paid in the last ten years to the genetic component that influences pain perception and opioid therapies, which may define the most suitable drug for any patient, both to provide adequate pain relief and to improve oncologic outcome [6].

In this narrative review we focus on mechanisms underlying genetic variation in opioid response; starting from the basics of opioids’ pharmacology, we present how genetic profile may determine pharmacokinetics and pharmacodynamics efficacy and safety of opioid medication, helping to choose the “ideal” medication (and adjust the dose) for cancer pain patients according to each individual’s characteristics. 

## 2. Methods

Our literature search was conducted in PubMed, using MeSH (Medical Subject Headings) and free-text terms. The Boolean operators “OR” and “AND” were used to help search terms for the main subjects. The first subject included terms relating to chronic pain conditions: “chronic pain” or “malignant” or “cancer” or “palliative care”; second, terms relating to pharmacogenetics: “pharmacogenetic” or “genetic” or “genomic” or “genotype” or “cytochrome P450 3A4/A5” or “cytochrome P450 2D6” or “CYP3A4/A5” or “CYP2D6” or “OPRM1” or “mu opioid receptor” or “ABCB1” or “UGT” or “UDP glycosyl transferase” or “COMT” or “catechol o amine methyltransferase”. Last, we searched for terms relating to the predefined opioids: “opioid” or “opiate” or “fentanyl” or “tramadol” or “codeine” or “morphine” or “oxycodone” or “tapentadol” or “methadone.”

Each of the groups of search terms was searched simultaneously with the “AND” Boolean operator to identify original research articles, reviews, and metanalyses published up to February 2020. The search was limited to articles published in the English language with human subjects. Other relevant articles were retrieved from the available papers.

## 3. Genetic Variation and Drug Response

### 3.1. Pharmacokinetic Phenotype (PK) 

**Figure 1:**
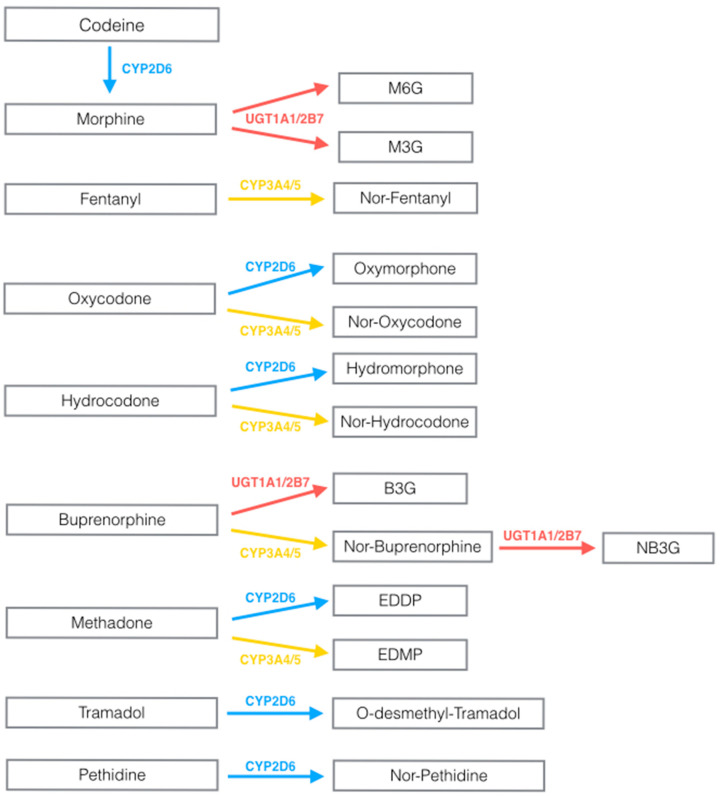
Schematic pharmacokinetics (PK) pathways of opioids commonly used in clinical practice. EDDP: 2-ethylidene-1,5-dimethyl-3,3-diphenylpyrrolidine; EMDP: 2-ethyl-5-methyl-3,3- diphenyl-1-pyrroline; B3G: buprenorphine-3-glucoronide; NB3G: norbuprenorphine-3-glucoronide. CYP: Cytochrome P; UGT: UDP-glucuronosyl-transferase. Colors are used to identify specific enzymes. Blue: CYP2D6, red: UGT, yellow: CYP3A4/5.

Opioids are mainly metabolized by the cytochrome P450 (CYP) system or by UDP-glucuronosyl-transferase (UGT) in the liver, which where therefore mainly investigated in the setting of opioids’ PK (Figure 1). 

#### 3.1.1. Cytochrome P-450 Genetic Variability and Opioid Pharmacokinetics 

Some opioids (see Table 1) are designed as pro-drug, and need to be converted to the active compound. Other opioids are already designed to be active and are deactivated to a lesser compound by the liver. 

The cytochrome P450 2D6 isoenzyme (CYP2D6) is primarily responsible for activation of opioid pro-drugs and deactivation of substrate opioid drugs [7]. The *CYP2D6* gene is characterized by extreme variability [8] that translates into a variable activity of the protein itself, causing a different response to drugs (including opioids) among individuals [9].

*CYP2D6* variability is classified in 4 phenotype groups: ultra-rapid metabolizer (UM), extensive (or normal) metabolizer (NM), intermediate metabolizer (IM), and poor metabolizer (PM [10]). 

Some case reports have described severe and even unexpected lethal adverse events in patients with CYP2D6 variants (especially UM) when treated with codeine and tramadol [11,12,13,14]. On the other hand, some studies have shown that *CYP2D6* may help in predicting the extent of analgesia in clinical patients. 

The wider part of the literature on *CYP2D6* deals with postoperative analgesia: available data suggest that genetic testing may be a valuable tool to improve opioid prescription in different surgical settings and for different molecules [15]. Less data exists for chronic pain, but there is no reason to think that similar advantages should not apply in chronic pain patients. Available data seem to confirm this assumption. A study showed that children having ineffective pain treatment of sickle cell crisis with codeine were more likely to have reduced CYP2D6 activity [16]; as well (in healthy volunteers) poor metabolizers experienced no analgesia after oxycodone administration in the experimental pain setting [9]. Further, in a cohort of 224 Italian patients with chronic low back pain (CLBP) treated with codeine or oxycodone, we showed that *CYP2D6* polymorphism was significantly (*p* = 0.018) associated with treatment outcome: reduced (CYP2D6*6) or absent (CYP2D6*9) CYP2D6 activity was correlated with lack of therapeutic effect, and ultra-rapid metabolizers showed an increased risk of side effects [17]. 

Recently, personalized opioid dosing algorithm based on *CYP2D6* variability has been proposed for clinician treating chronic pain, to improve safety and efficacy [18,19]. This study is an important proof of concept: patients on chronic pain (*n* = 375) and scheduled to receive tramadol/codeine were assigned to standard care (meaning that opioid prescription was not genetically-guided) or to a CYP2D6-guided prescription. In the latter group, CYP2D6 phenotypes were assigned based on genotype and CYP2D6 inhibitor use, with recommendations for opioid prescribing according to this phenotype. IM/PMs had greater improvement in the CYP2D6-guided versus usual care arm (*p* = 0.016); furthermore, clinically meaningful reduction in pain was more often observed (24% versus 0%) when patients were prescribed according to CYP2D6 phenotype rather than usually done. On the other hand, NM didn’t show any difference when genetic-guided approach was pursued instead of usual (*p* = 0.54). This trial demonstrated successful implementation of CYP2D6-guided opioid prescription and improved pain control in patients theoretically more prone to benefit of this pharmacogenetic approach (IM/PMs prescribed with tramadol or codeine) [20].

Surprisingly, very few studies deal with chronic cancer pain. With not much data available, the evidence of genetics on clinical outcomes is controversial. A recent study in cancer patients shows that *CYP2D6* genotype impacts on plasma concentrations of tramadol and its demethylated metabolites, as well as drug tolerability [21]. On the other hand, previous data evidenced that although *CYP2D6* genotypes had clear effects on the pharmacokinetics of opioids (namely oxycodone), no difference was found between the class of metabolizers in pain intensities, nausea, tiredness, and cognitive function [22]. 

This lack of clinical association may be linked to several factors, cachexia being one of the major ones. Cachexia is associated with abnormalities in a body’s homeostasis that reflect a chronic inflammatory status, and has been demonstrated to impact on oxycodone metabolism in different studies [23,24]. Further, the abovementioned conflicting results were retrieved for oxycodone, whose metabolism is only 20% related to CYP2D6 (80% being associated with CYP3A4 [25]). 

Taken together, these data suggest that *CYP2D6* profoundly impacts opioids metabolism, but differences between opioids may exist. As well, differences in clinical effects should consider other factors (specific of the oncologic patient, i.e., cachexia and organ failure) that may influence opioid metabolism. These motivations strongly recommend new studies in this specific population, where indications drained by postsurgical setting and benign pain may apply differently and may justify discrepancies in clinical efficacy/safety. Further data from dedicated studies, focusing on specific opioids and analyzing cancer-specific variables may better guide CYP-related opioid prescription.

Other relevant genes for opioids are cytochrome P450 family 3 subfamily A members 4 and 5 (*CYP3A4* and *CYP3A5*) [26]. They are especially relevant for inactivation of synthetic opioids [26] (see Figure 1). Genetic variability in these genes is lower than *CYP2D6* [27,28,29] and they may therefore result less relevant for screening purposes in clinical practice [30]. However, large patients’ cohorts showed an effect of such genetic variations on different opioids in the clinical practice, both in postoperative and chronic cancer patients (see Table 2). Allelic mutations in *CYP3A4* and *CYP3A5* encode for decreased enzymatic activity [29], which showed to influence transdermal fentanyl metabolism. However, other clinical factors like CYP3A inhibitors and variables relating to liver and kidney function (serum albumin, glomerular filtration rate, kidney disease, body mass index—BMI) were associated with metabolic ratio, and accounting for a minor part only (14%) of its variability [31]. As for *CYP2D6* these variables might be considered in the future, since their relationship to efficacy and adverse effects may aid in improving the safety and efficacy of opioids [31]. Concerns remain, but studies with high sample size are required, because initial small-populations trials gave inconclusive results.

In summary, CYP2D6 isoenzyme is involved in opioid PK and is able to influence analgesic effect and adverse events in patients with cancer pain. Opioid pro-drugs activated by CYP2D6 fail in providing analgesia in PMs (higher doses may be required), while drugs that are deactivated by CYP2D6 may be dangerous in the first. On the contrary, UMs should avoid pro-drugs because of the documented possibility of lethal side effects, while inactivated drugs may be ineffective. CYP3A4/5 may also impact on opioid response. Relative influence may vary according to the type of opioid and (most relevant) to other features (cachexia, liver and kidney function) peculiar for the clinical scenario of cancer patients. 

#### 3.1.2. UGP Genetic Variability and Opioid Pharmacokinetics (PK) 

UDP-glucuronosyl-transferase (UGT) 2B7 is the cornerstone for morphine metabolism: morphine is glucuronated to morphine 6-glucuronide (M6G) and morphine 3-glucuronide (M3G) [47]. M6G has been shown to be a potent analgesic (analgesic properties of morphine are enhanced by the action of M6G), while M3G decreases the analgesic activity of morphine and M6G [48]. Pharmacokinetic profiles of morphine and its metabolites could help clinicians in optimizing individual therapies and tailor the dose to individual needs in cancer patients [49].

As for Cytochrome P-450, genetic polymorphisms may ideally translate into a variable activity of the enzyme and of morphine PK. However, studies highlighted their minor clinical significance. Polymorphisms in *UGT2B7* in both the other coding and regulatory region were studied in different cohorts on long-term morphine therapy [50]. Overall, no association was found between UGT2B7 genotype and haplotype and the morphine/morphine glucuronide ratios in cancer patients with normal renal and hepatic function [51]. Even when some haplotype was associated in vitro with reduced function, no association was replicated in the in vivo population [52]. These studies concluded that the variability in morphine and its metabolites is mainly attributable to other factors.

A further study in cancer patient established that the genotypes or allelic frequencies of various *UGT2B7* polymorphisms were not significantly (*p* > 0.5) associated with responders or non-responders [34], with no association between genotype and the serum concentrations of morphine or its metabolites [34]. Recently, two haplotypes were identified in 759 Caucasian cancer patients from the EPOS study in *UGT1A1/UGT1A8* showing weak associations with lower morphine glucuronide to morphine ratios after oral administration of morphine [53].

Surprisingly, polymorphisms in *UGT2B7* have been significantly associated with morphine-induced adverse effects in a study on Japanese cancer patients under oral controlled-release morphine: the frequency of nausea was significantly associated with the UGT2B7*2 (*p* = 0.023), which appears to have a protective effect [54]. However, larger amounts of data need to confirm this finding.

To summarize, *UGTB7* variability seems not to determine clinical variations in patient’s response to morphine. Some effect may be exerted on side effects, but very few data are available. However, molecules with UGT-based metabolism (morphine, hydromorphone) may be a valuable choice for patients with CYP variants or concomitant medications that contraindicate the use of other opioids.

### 3.2. Pharmacodynamic Profile

#### OPRM1 and COMT Genes Variability Influences Opioid Pharmacodynamics

The μ opioid receptor (OPRM) is the main target of both endogenous- and clinically-used opioids primarily involved in drug dependence and opioid-induced respiratory depression [55].

The μ1 opioid receptor gene (*OPRM1*) codes for this receptor. A functionally important variant is the mostly studied (termed A118G - rs1799971 [56,57]. 

Results in experimental pain settings indicate that the G allele is related to a lower threshold of pain perception. Having at least 1 copy of the G allele (AG or GG) is associated with lower pain threshold, and higher opioid consumption in post-operative patients as well [56]. Thus, this common polymorphism found in *OPRM1* could render a patient less sensitive to opioid analgesic effects, and more prone to dependence [56,58]. Some metanalysis on postoperative pain in adults showed that the G variant had higher opioid requirements and a lower risk for adverse events [32,59,60]. The 118A_G variant seems to be a potential biomarker in clinical practice for postoperative pain, potentially predicting opioid requirements, short and long-term pain, and cancer outcome [6]. 

However, results on chronic cancer and non-cancer patients are conflicting [39]: some studies showed that patients with the G allele are prone to have more pain and higher morphine requirements (with no differences in side effects), while other studies failed in demonstrating any association (see Table 2). Even though some promising results have been reported, the details on *OPRM1* may be more complicated: we need to elucidate the magnitude of its effect on opioid response in combination with other genetic variants (e.g., *CYP2D6, COMT,* or *ESR1*) [61] and the different effect per type of opioid in the dedicated setting of chronic pain treatment.

Catechol-O-methyltransferase (*COMT*) gene is one of the most frequently analyzed genes in the pain scenario. This gene codes for COMT enzyme, which is involved in numerous physiological functions (including pain perception) [62,63,64,65] by regulation of μ−opioid receptor (MOR) expression [66,67]. 

Three functional haplotypes have been defined, i.e., low pain sensitivity (LPS = Met/Met), average pain sensitivity (APS), and high pain sensitivity (HPS = Val/Val) [64]. More recently, the LPS haplotype has been associated with the highest pain score and opioid consumption in the postoperative pain setting, while Met158Met-genotyped patients required less opioid for both cancer pain [38,68,69,70] and postoperative pain or showed fewer side effects [71,72,73,74,75,76]. 

Different studies have been conducted to investigate the correlation between *COMT* variations and opioid requirements in perioperative pain. They have been recently pooled in a metanalysis, which failed to detect any association with postoperative pain and opioid requirements in the perioperative setting [77]. Reasons for this may rely on underpowered samples, as well as on the traditional approach of testing single SNPs rather than combination of them. As a matter of fact, a recent trial found that combinations of genetic variants within *OPRM1, COMT*, and *ESR1* genes better explain variability in morphine consumption than single genetic variants after major abdominal surgery [61]. Rapid development of whole genome sequencing enables information on all genetic modifications that may affect analgesic response, and may help understanding the effect of multiple genetic variations on postoperative pain [77].

In the context of chronic cancer pain, *COMT* variability has been associated with morphine requirements in different studies, including the pediatric population [78]. However, while some studies documented positive correlations, some others provided conflicting results [53]. Since many studies have low sample sizes and may be underpowered according to the type of outcome analyzed, results should be interpreted with caution. Some of the papers proving positive results frequently include less than 100 patients [68,78], while the population needed to detect a proper effect size for clinical decision-making (i.e., a clinically meaningful change in opioid requirements) may approximately be of 550 patients per group [53]. As a matter of fact, replication cohorts of >1000 patients failed to confirm any association between gene variations (including *COMT*) and opioid requirements [53]. Thus, further studies with higher samples and multiple testing are required to solve the open questions on *COMT* variability. 

*COMT* may influence opioid response with other-than Val158Met variability. Rakvag et al showed that a specific haplotype including 11 *COMT* SNPs (that is mostly represented in the Caucasian population) was associated with less morphine consumption than non-carriers did [70]. This approach based on multiple SNPs and haplotype construction may help in understanding the variability in opioid consumption. This approach may help overcome limitations associated with most of the studies, i.e., sample sizing and the SNP-by-SNP approach (variations on 1 or few regions are analyzed), which limits the interpretation of clinical outcomes, where a number of biological mechanisms and a plethora of genes are involved [53].

In summary, *OPRM1* and *COMT* gene variations are involved in both pain sensitivity and opioid response. Cancer patients with at least one A allele (*OPRM* – A118G variant) and a Met allele (*COMT* Val158Met) seem to experience less pain and more analgesia, with less side effect. Other genes involved in drug transport and metabolism are likely to have an effect on clinical response, alone or in combination. 

## 4. Discussion

Pain is a main concern for most of patients with cancer: pain is limiting for their quality of life and also has a huge impact on their outcome [79]. Opioids are the most frequently prescribed drugs for cancer pain management. Despite their wide adoption, concerns still remain about both their efficacy and safety. Some patients still suffer for incomplete pain relief and side effects of are an issue in the short- and long-term.

One of the major contributors in determining pharmacologic response (including opioids) is the genetic background, which is different among patients and can influence several aspects of drug response (pharmacokinetics) and sensitivity (pharmacodynamics). Theoretically, mapping the inter-individual variability in genes involved in opioid response is the “Holy Grail” for pain physicians. Knowing how a patient may react to a specific drug may actually be the main step towards a more effective “personalized” therapy. Pharmacogenetics has the specific endpoint of understanding those genetic variations, in order to tailor specific treatments to each and every patient. This is even more important in cancer patients: they are usually multi-pharmacologic, more prone to drug–drug interactions and potential adverse events. The “trial-and-error” approach is ineffective, dangerous, costly, and causes delays in effective care: pharmacogenetics and personalized medicine may help in overcoming this obsolete approach, using genetic tests to assess each patient’s risk for adverse events and the likelihood to positively respond to a given drug, thereby allowing an “informed” drug selection. 

In such a scenario, different genetic variants have been investigated for many aspects of opioids, including both PK and PD features. In a clinical purpose, it is even more important for prescribers to understand how genetic variants affect efficacy and safety, i.e., how each variant and their combinations affect pain relief and the development of side effects. Testing a patient for this purpose may help changing prescriptions to improve therapeutic profile, determining the most effective drug at the safest dose. 

In the field of PK (which basically explains how the drug is metabolized by the body) the main actor is the cytochrome P 450 family, which is involved in liver metabolism of many drugs (including opioids). Different isoforms are active in opioid metabolism, but CYP2D6, 3A4, and 3A5 are the main (or at least those who have been mostly investigated).

Testing CYP2D6 in the clinical setting provided interesting data; although less of them come from the specific scenario of cancer pain management, they suggest that CYP family testing may help better opioid prescribing. It’s probably too early to tell if we can screen patients to specifically identify those who will respond better and with lower doses, although studies to genotype patients and guide oxycodone prescribing are in place [18], and a recent trial showed that *CYP2D6*-guided opioid therapy improves pain control in *CYP2D6* intermediate and poor metabolizers [20]. In any case, CYP testing may help in screen patients with extreme phenotypes, i.e., those prone to treatment inefficacy or serious adverse events [80]. 

A recent paper has even proposed an alternative to classic nomenclature that is more practical for clinician [7]. This system classifies 3 different phenotypes for the *CYP2D6* gene (functional, sub-normal, dysfunctional). With this approach, patients with dysfunctional alleles should be treated with caution: CYP2D6 substrate drugs (including opioids) should probably be avoided since they may result in poor efficacy and/or increased adverse events (see Table 1). Specific indications already exist for some opioids. The Clinical Pharmacogenetic Implementation Consortium (CPIC) has published clinical recommendations for codeine dosing based on *CYP2D6* genotype [81]. Both *CYP2D6* UMs and PMs should avoid codeine: in the former group a rise in morphine formation will increase toxicity, while in the latter no analgesic effect is weaker (due to reduced morphine formation). Literature provides less evidence for other opioids; caution should especially be taken with the use of tramadol, oxycodone, and hydrocodone.

The Dutch Pharmacogenetics Working Group (DPWG) has produced other relevant guidelines [82]. Even though (in general) some difference is retrievable between guidelines, efforts have been made to analyze and harmonize them [83]. In the specific case of opioids (namely codeine) the CPIC and DPWG recommendation fit well and complement each other; recently, they were the bases for the development of the pharmacogenetics-guided passport, i.e., a panel of actionable germline genetic variants for pre-emptive pharmacogenetics testing [84]. Furthermore, a recent study by Gammal et al. has demonstrated the successful application of this genetics-driven approach in patients. The authors describe the implementation of pharmacogenetics-based codeine prescribing that accounts for CYP2D6 metabolizer status. Clinical decision support was implemented to guide codeine prescribing in CYP2D6 UM and PM (recommending against codeine for patients with these high-risk CYP2D6 status). None of them were prescribed with codeine. Using genetics to tailor treatments had an important effect by limiting codeine use to patients who could safely receive and benefit from it [80]. This is another proof-of-concept for safer and effective use of opioids when prescriptions are tailored on genetic background.

CYP2D6 status is also important when other medications affecting the same/other pharmacokinetic pathways are concomitantly administered. Several drugs have common metabolic pathways and combining them can alter drug kinetics and lead to lethal consequences in patients with existing comorbidities or a pre-existing UM status.

Further, since CYP450 family affects many drugs and their metabolism, whenever patients display abnormal reactions to these drugs, a genetic contribution should be always considered. Once investigated, such a contribution may unravel an unexpected abnormal response to opioids.

In these cases, or whenever drug–drug interaction may be suspected, molecules with different metabolic pathways may be a better choice. Since the available data on *UGT* genetic variability failed to demonstrate any important association with opioid response [66], morphine and hydromorphone (that are metabolized by UGT and not by CYP family members) are a good choice to reduce variability in opioid efficacy and the development of side effects, and may not predispose to interference with other compounds with different metabolic process. [85], as well, is a good alternative. Although it is similar to tramadol, Tapentadol is not a pro-drug and does not rely on metabolism to produce an analgesic effect (no CYP interference) [85]; this makes it a useful molecule for patients who do not respond to more commonly used opioids due to their genetic disposition. Tapentadol is an opioid and a noradrenaline re-uptake inhibitor (similar to tramadol): its pharmacogenetics may be primarily deduced from known associations of *OPRM1*, *KCNJ6,* and COMT gene variants [86].

Pharmacogenetics is a major tool for clinicians when approaching to PD of opioids. In this context, more data are available than for PK, especially for *OPRM1* and *COMT* genes. Many open issues remain that need to be fixed in the future, but it is honest to affirm that identifying patients with some specific variants of these two genes may help in better opioid prescription. 

Patients with *OPRM1* A/A genotype and patients with *COMT* Met/Met genotype have been invariably associated with a more favorable phenotype, also in specific cancer patients’ populations: less pain sensitivity, better response to opioids with lower consumption, less side effects [35,36,39,66]. These results have been mainly shown in patients prescribed with morphine, but no data seem to exclude that they can be replicated with other types of opioids (Table 2). 

Further, since opioids are immunosuppressive and affect the endocrine system [3], they might directly and/or indirectly influence long-term cancer recurrence. MOR is a major element underlying (and connecting) pain, endocrine system, and immune function: OPRM1 might be used in the near future to customize opioid therapy, avoiding not only opioid side effects but also disease progression [6]. 

As for CYP testing, pharmacogenetics may help screening patients prone to ineffective pain relief and adverse events. The same authors proposed a simple classification (as for *CYP2D6*) for clinical management of patients with *OPRM1* variants based on 3 allelic variants: Normal, sub-functional, dysfunctional [7]. When *OPRM1* is subnormal or dysfunctional, the patient is less likely to benefit from opioids, and more likely to experience side effects and dependence [7]. This approach would also change the prescribing approach whenever a patient does not get effective pain relief: escalating opioids, as well as adding other opioids may not increase analgesia if the patient has a dysfunctional *OPRM1* status. Relying on non-opioid drugs or alternative therapies may be better, in this case. 

Of course, combining data on pharmacokinetics and pharmacodynamics may further help in stratifying patients more prone to receive opioids. Of note, the list of gene variants affecting opioid metabolism is not restricted to *OPRM1* and *COMT*: several other genes have been identified that code for the cell’s channels and transporters involved in opioid signaling that finally reflect in different patient’s response to a given drug (see Table 2). These other genes act alone or in combination and may lead to different opioid consumption and efficacy, as well as to a different side effects profile. When combined, all of these variants may configure some panels for genetic testing in patients prescribed with opioids.

Since different genes regulate pain and analgesia, approaching pharmacogenetics with a multiple SNPs approach may be promising [53]. To the same extent, genome-wide association studies (GWAS) would be required to identify all genetic factors involved in pain and opioid response, being helpful for better prescriptions. GWAS found new SNPs, whose mechanisms might not seem clear, but associated with opioid response. Recently a GWAS on more than 1000 cancer patients identified eight SNPs significantly associated (*p* < 1.0 × 10^−3^) with pain relief. However, rs12948783 of the *RHBDF2* gene showed the best statistical association [87]. This genetic variant rs7104613 is located on a gene encoding for a cell-adhesion protein that promotes the attachment of spinal cord and sensory neuron cells and the outgrowth of neurites in vitro. This result underlines the involvement of genes related to transmission of nerve impulse, and not opioid pharmacology, in mediating the main modulatory effect of genetic variability on the perception of pain. 

A recent analysis on 4 different populations with thousands of patients identified a variant on chromosome 15, rs12442183, near *RGMA*, associated with opioid dependence (*p* = 1.3 × 10^−8^) [88]. *RGMA* encodes repulsive guidance molecule A, which is a central nervous system axon guidance protein. GWAS (Genome—Wide Association Studies) unraveled new lead into our understanding of pain pathophysiology [89], confuting the traditional attention paid to genes involved in opioid pharmacology only. Other GWAS data suggest that opioid therapy outcome more likely results from interaction of both pathways [90], also because some of these “new” genes function is still unknown or uncertain. However, GWAS studies provide promising targets that might contribute to personalized medicine.

Better prescribing and better patients’ management may also have a pharmaco-economic impact. For example, 70% of cardiac drugs showed a different response or a different risk of adverse effects based on genetics, supporting the idea that having this information before prescribing is warranted [91]. The same was also proven for warfarin treatment [92]. Advances in pharmacogenetics may optimize patient outcomes while reducing costs associated with inappropriate or unsafe prescribing [93]. Even if a few studies are available, there is increasing evidence that pharmacogenetics may help reducing healthcare costs. Direct costs may be influenced by better drug’s biological activity, while indirect costs may be lowered by the prevention of side effects (especially addiction in non-cancer patients [94]). Finally, pharmacogenetics could be useful to predict drug–drug interactions [95,96], really common in cancer patients because of the concomitant intake of several different drugs (especially in slow or ultra-rapid CYP4502D6 metabolizers).

On the other hand, costs for obtaining and interpreting samples, as well as counseling patients should be accounted. However, broader data and more knowledge, combined with refined and cheaper testing, may help overcoming the obsolete “trial-and-test” philosophy, improving patients’ outcome and impacting on healthcare costs [97]. Some proof already exists: a recent trial including pharmacogenomics information in the healthcare pathway of inpatients with high risk profile, reduced readmissions by 52%, emergency department visits by 42% and mortality by 85%, with an estimated cost savings per patient of $4382 in just 60 days [98].

The same concept may be applied to pharmacologic development in industries [99], which may save lot of money during drug development. It is currently difficult to estimate the actual effect of introducing pharmacogenetics in the clinical day-case scenario and the most relevant tests to be used, but genetic testing may be extremely useful in overcoming the important rate-limiting step of costs in providing adequate therapies. 

In the meantime, when a genomic-oriented medical approach is pursued, ethical concerns rise (as well as with any “omics” approach), especially in the field of pain medicine. Genomics (and other “omics”) technologies produced not only a deep change in biomedical research, but the potential to discover large amounts of possibly important incidental information is an issue, as well as physicians’ obligations arising from this type of the research, protection of the included individuals and personal data, and communication of such research to patients and the community [100].

In the specific context of opioids, pharmacogenetics may exacerbate inequalities in the delivery of healthcare [101]. Genetic testing may identify patients more prone to effective analgesic response, but also those at high risk of opioid adverse events, such as abuse and addiction. 

This information may lead to different treatments covered by health insurance companies, since patients more prone to have “poor profile of response” would be unraveled before treatment [101]. 

Hence pharmacogenetics could raise the following questions: who decides to perform the test: patients? Care providers? Insurance company? Once a genetic-driven prescription is pursued, how the needs of insurance companies will fit with the variability of the clinical scenario?

Finally, pharmacogenetics information can vary according to racial or ethnic origin. Discovering a genetic variant that influences a medicine’s response in a particular ethnic group, may lead to the design of medications for a specific ethnic group or for an ethnic group most likely to be able to afford the medications [102]. This would be a concern if the ethnic group more prone to a “good response” to a drug is not equally able to afford it.

Once the concept of pharmacogenetics becomes mainstream and knowledge will increase in patients as in care physicians, one may lie or forge his profile to obtain specific drugs (and abuse of them) [103]. This would be the nemesis of pharmacogenetics: born to improve outcome, it would be the cause of abuse and diversion. 

Of course, these are potential scenarios that may configure or not, but they should be kept in mind when evaluating the potential implications of genetic profiling for opioid prescriptions, especially in cancer patients, in which ethical concerns are strictly related to life expectations.

## 5. Conclusions

Pharmacogenetics to guide opioid prescription is not a myth, but likely to become a reality. Even though many concerns remain in the complex scenario of genetic variability in the PK and PD of opioids, some candidates have been identified to have a specific role. Of course, the impact of their role should be better defined, but it is straightforward to think that some genetic variants may already be used to select groups of patients with different clinical response. Initial studies on the pharmacogenomics-guided approach to opioid prescription are promising: they already led to higher efficacy and reduced side effects, and may apply to all opioids.

Limiting factors still exist to the widespread introduction of genetic testing in opioid prescription dealing with costs, selection of patients, and ethical concerns. However, as far as our knowledge will increase and new data will confirm the validity of genetic profiling, these limits may be overcome. The complexity of mechanism involved in opioid response may not be fully elucidated in the near future, but even refined knowledge on a few genes may be the cornerstone for the diffusion of genomic-guided opioid prescription to improve a patient’s outcome.

## Figures and Tables

**Table 1:** Expected different response to opioid therapy in patients with different gene phenotype.

**PRO-Drug**	**Active Compound**	**Poor Metabolizers**	**Ultra-Rapid Metabolizers**
*Codeine*	Morphine	↓ efficacy	↓ efficacy ↑ AE
*Oxycodone*	Oxymorphone	↓ efficacy	↓ efficacy ↑ AE
*Hydrocodone*	Hydromorphone	↓ efficacy	↓ efficacy ↑ AE
*Tramadol*	O-desmethyl-tramadol	↓ efficacy	↓ efficacy ↑ AE
**Drug**		**Poor Metabolizers**	**Ultra-Rapid Metabolizers**
*Pethidine (Meperidine)*	Nor-pethidine	↑ AE	↓ efficacy
*Morphine*	M6G/M3G	↑ AE	↓ efficacy
*Methadone*	EDDP - EDMP	↑ AE	↓ efficacy

EDDP: 2-ethylidene-1,5-dimethyl-3,3-diphenylpyrrolidine; EMDP: 2-ethyl-5-methyl-3,3- diphenyl-1-pyrroline; AE: adverse events; M6G: morphine-6-glucoronide; M3G: morphine-3-glucuronide.

**Table 2:** Commonly used opioids and genetic variants reported to modulate response. Bold text refers to studies in cancer patients.

Gene (protein)	Drugs Involved	Function	Available Data
*CYP2D6* (Cytochrome P450 2D6 isoenzyme)	Codeine Oxycodone Hydrocodone Methadone Tramadol	Activation of prodrugs De-activation of the active drug	**CYP2D6 genotype impacts on plasma concentrations of tramadol and its demethylated metabolites, as well as drug tolerability** [21]. **CYP2D6 genotypes had clear effects on oxycodone PK but lack of clinical difference (pain/adverse events) between classes of metabolizers** [22].
*CYP3A4* (Cytochrome P450 family 3 subfamily A members 4)	Fentanyl Sufentanyl Alfentanyl Oxycodone Hydrocodone	Inactive metabolites are formed via N-de-alkylation Oxycodone: 80% of this drug is converted by CYP3A4 to the non-active metabolite nor-oxycodone	*22 allele mutation encodes decreased CYP3A4 enzymatic activity [29]. Fentanyl (n = 620) showed that genetic variability in CYP3A4*22 influenced transdermal fentanyl metabolism [31]. CYP3A4*1G carriers consumed less opioids [32]
*CYP3A5* (Cytochrome P450 family 3 subfamily A members 5)	Fentanyl Sufentanyl Alfentanyl	Inactive metabolites are formed via N-de-alkylation	Most frequent genetic variant in white individuals is the inactive CYP3A5*3 allele 80% of white individuals are homozygous carriers of this allele = do not express (non-expressers [28]) CYP3A5 expressers who carry at least one *1 allele may require lower doses CYP3A5*3 allele = more adverse effects [33]
*OPRM1* (μ-opioid receptor)	Opioids		**OPRM1 SNPs were not significantly different in patients with higher pain and side effects** [34] **OPRM1 SNPs were associated with increased opioid requirements in cancer patients** [35,36,37] **OPRM1 A/A + COMT Met/Met genotype = lower dose of morphine** [38] OPRM1 variants were associated with increased opioid requirements for postoperative pain, while other gave opposite results or failed to demonstrate any association in the postoperative period [39]
*COMT* (Catechol-O-methyltransferase)	Opioids		**COMT Val/Val genotype = higher dose of analgesics** [37] **OPRM1 A/A + COMT Met/Met genotype = lower dose of morphine** [38]
*ABCB1* (ATP binding cassette subfamily B member 1)	Fentanyl Oxycodone Morphine	Efflux pump in the intestine and at the blood–brain barrier	The 3435C > T SNP is mostly studied In 3435TT genotyped individuals = higher expression = higher morphine concentrations in the cerebrospinal fluid [40]. Lower opioid doses might be needed in these patients (risk of side effects on normal dosages), but results are contradictory [32] Morphine equivalents decreased in a gene dose-dependent manner with the variant ABCB1 3435C > T [41].
*ABCC3* (ATP-binding cassette subfamily C member 3 transporter)	Morphine	Transporter involved in the efflux of M3G and M6G from the liver to the bloodstream	The promoter SNP-211C>T (rs4793665) was associated with decreased ABCC3 mRNA expression [42] Children—postoperative pain: 211C > T polymorphism was associated with lower M3G and M6G plasma concentrations [43]
*SLC22A1* (Organic Cation Transporter 1)	Tramadol Codeine Morphine	Present on the sinusoidal membrane of hepatocytes. Uptake of positively charged compounds at physiological pH (morphine, active metabolite of tramadol)	Tramadol: increased plasma concentrations of O-desmethyltramadol [44] and lower tramadol consumption during the first 24 h after surgery [45] Increase of 56% in morphine AUC after codeine administration and lower clearance in children [43,46] These genetic variants could explain why children with a white background experienced more adverse events than African American children, in whom the variant alleles are less frequent [43]. SLC22A1deficient alleles may be especially relevant for patients carrying the CYP2D6 ultra-metabolizing status
*KCNJ6* (G protein–activated inwardly rectifying potassium 2 channel- GIRK2)	Opioids	The encoded channel is one of the downstream signaling effectors of the MOR.	Several SNPs in the KCNJ6 gene were related to opioid response but negative findings have been published as well.

AUC: area under the curve; SNPs: Single Nucleotide Polymorphisms; MOR: μ-opioid receptor.
